# Penile Inversion Vaginoplasty: An Evolving Technique

**DOI:** 10.7759/cureus.68949

**Published:** 2024-09-08

**Authors:** Samyd S Bustos, Maria Rios-Sanchez, Vahe Fahradyan

**Affiliations:** 1 Plastic and Reconstructive Surgery, Mayo Clinic, Rochester, USA; 2 Medicine, Universidad Central Del Caribe, Bayamon, PRI

**Keywords:** feminization, gender-affirming surgery, penile inversion vaginoplasty, transgender, vaginal reconstruction

## Abstract

Penile inversion vaginoplasty (PIV) is the most common surgical technique used in “gender-affirming bottom surgery.” During this process, penile tissue is used to create a functional neo-vagina, allowing the individual to experience a more aligned physical manifestation of their “gender” identity. In this technical note, we describe the steps and nuances used by the senior author to ensure reliable aesthetic and functional outcomes, contributing to the overall well-being and satisfaction of transgender patients.

## Introduction

“Gender-affirming vaginoplasty” is a surgical intervention designed to create the female vulva and vaginal canal in individuals assigned male (AMAB) at birth who are “gender” diverse [1-3]. Since its initial description, myriad surgical modalities have emerged for the reconstruction of the vaginal anatomy [2,3]. Techniques encompass penile inversion utilizing penoscrotal flaps and skin grafts, extragenital skin grafts, peritoneal flaps, grafts, and intestinal procedures [2-6].

The term “penile inversion vaginoplasty” (PIV) specifically refers to the technique wherein penile skin is inverted and advanced into the neo-vaginal space, a technique pioneered by Gillies and Millard in 1957 and subsequently refined and popularized by Dr. Georges Burou [2]. Despite subsequent modifications, this approach continues to be the most common procedure in “gender-affirming bottom surgery,” widely regarded as the gold standard by many surgeons specializing in “gender-affirming bottom surgeries” [5].

PIV has emerged as the most extensively researched technique in recent years, rendering it the foremost “evidence-based” approach for “gender-affirming vaginoplasty” [5-7]. In a comprehensive systematic review of 2021, a total of 4680 cases were represented; 39 (75%) studies used the penile skin inversion technique with or without scrotal graft, and 11 (21.2%) studies used bowel pedicle flaps [5]. While complications occur, most are self-limited and can be treated conservatively or with minor interventions [2,4,7]. Minor complications include granulation tissue formation, intravaginal hair growth, delayed wound healing or disruption, aesthetic concerns, and introital stenosis [2]. Other less common complications include stenosis of the neovaginal canal, rectovaginal fistula, and pelvic floor dysfunction [8,9].

In the realm of “gender-affirming surgery,” important outcomes include patient satisfaction, functional outcomes, quality of life, and the alleviation of “gender” dysphoria [2,8]. As medical and surgical professionals caring for this patient population, we are responsible for advancing research endeavors to optimize surgical techniques for our patient's benefit. In this technical note, we aim to describe the surgical nuances and changes in practice of the senior author’s surgical technique (VF) for PIV. This method involves utilizing scrotal skin as a full-thickness graft and the tunica vaginalis, which forms the structural basis of the neo-vaginal lining.

## Technical report

Preoperative considerations

Developing a multidisciplinary team that can address and fulfill the patient's needs is crucial. According to the most recent Standard of Care guidelines of the World Professional Association for Transgender Health (WPATH), this holistic team comprises mental health specialists, surgeons, endocrinologists, internists, social workers, and physical therapists [1]. It is essential that the surgeon reviews options, risks, and complications when informed consent is provided. In the perioperative period, we require patients with diabetes to have an A1C below 7%. At our center, there is no body mass index (BMI) cutoff for PIV, but we strongly recommend that patients have a BMI of 35 kg/m^2 ^or lower. This may vary per center [5-7]. No routine imaging is recommended. Preoperative labs such as complete blood count, metabolic panel, and electrocardiogram are done. Patients typically undergo permanent hair removal in the areas used for the neo-vaginal lining, or the hair follicles are cauterized at the time of skin harvest.

In our institution, patients are not routinely required to suspend hormone therapy before surgery. Our endocrinologist's team manages their care, including clinicians from the Transgender and Intersex Specialty Care Clinic (TISCC). The team reviews medical records for each patient to consider comorbidities, risks, and individual needs. Our preference on deciding whether to suspend or continue hormone therapy and anticoagulation is made on a case-by-case basis, tailored to each patient's specific circumstances [10]. In our experience, we have not identified a difference in outcomes, and in the surgeon's practice, we have never suspended hormone therapy before surgery. 

Surgical technique

**Figure 1:**
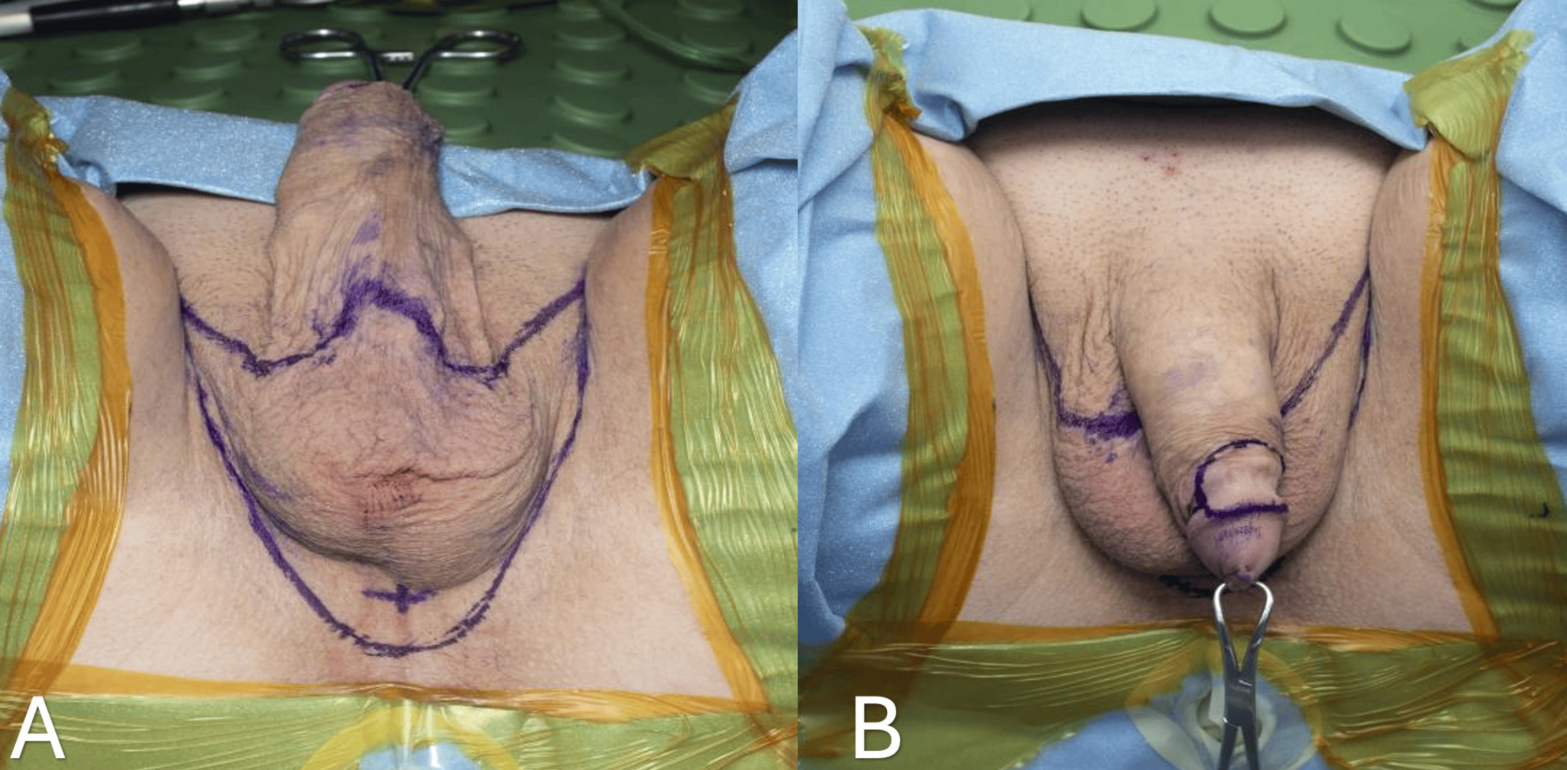
Butterfly incision is marked for scrotal skin graft harvest (A). A 2 x 0.5 cm flap is marked at the dorsal corona of the glans penis (penis crown), and a 2 x 3 cm dorsal penile skin flap is designed that is attached to the coronal edge (B).

This comprehensive procedure encompasses a series of steps, including scrotal skin graft and tunica vaginalis harvest, orchiectomy, penectomy, creation of the neo-clitoris, urethroplasty, creation of the neo-vaginal space, and bilateral labioplasty. The patient is placed in a lithotomy position. A transurethral resection (TUR)/urological surgical drape is used to allow sterile rectal examination during the procedure. Next, a butterfly incision is marked for scrotal skin graft harvest. This typically involves the entire scrotal skin and part of the perineal skin. It extends from the base of the penis to a point 1.5 to 2 cm anterior to the anal canal, aiming to prevent any potential injury to the anal sphincter. The lateral edges of the incision are positioned just medial to the inguinal creases. Next, a 2 x 0.5 cm flap is marked at the dorsal corona of the glans penis (penis crown), and a 2 x 3 cm dorsal penile skin flap is designed that is attached to the coronal edge (Figure 1). These will become the neo-clitoris and inner lining of the clitoral hood, respectively.

**Figure 2:**
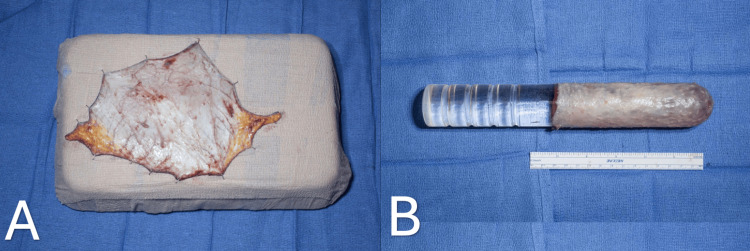
Processing of the graft, excision of residual fatty tissue, and cauterization of hair follicles with needle electrocautery (A). Tabularization of scrotal skin graft (B).

Scrotal skin graft preparation is a meticulous procedure involving the excision of residual fatty tissue and cauterizing hair follicles with needle electrocautery. This optimizes the survival of the neo-vaginal lining and reduces the risk of hair growth in the vaginal canal. The full-thickness scrotal skin graft is positioned on its dermal side up on the largest vaginal dilator and is tubularized (Figure 2). When the skin graft is insufficient, a tunica vaginalis mucosal graft is placed radially on the dilator, tubularized, and sutured to the scrotal skin graft. 

**Figure 3:**
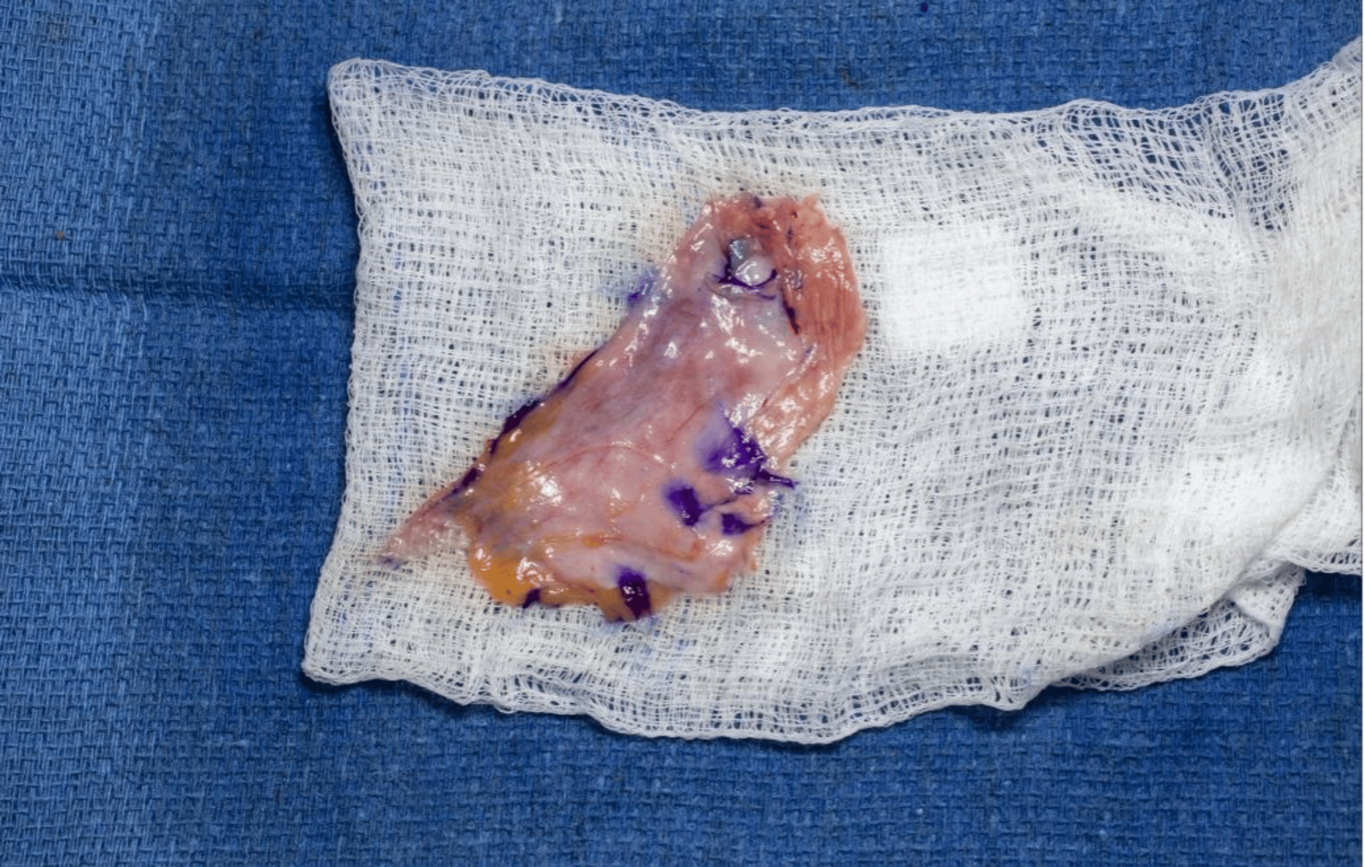
Harvested tunica vaginalis graft.

A simple orchiectomy is then performed, during which testes with their spermatic chord are exposed up to the external inguinal ring, ligated, and then cut, allowing it to retract into the inguinal canal. We prefer double ligation of the spermatic cord with a large suture as a precaution to potentially retrieve the ligated structures, as any bleeding could be challenging to manage once the cord is retracted into the inguinal canal. We then harvest the tunica vaginalis and preserve it in case we need to use it if the scrotal skin graft does not suffice (Figure 3).

Some authors recommend closing the external ring to prevent hernia formation [11]. Nevertheless, it is critical to note that the ilioinguinal nerve passes closely and that any inadvertent injury to this nerve can result in neuroma formation and chronic pain. We do not routinely close the external inguinal ring and have not encountered any hernia cases thus far.

**Figure 4:**
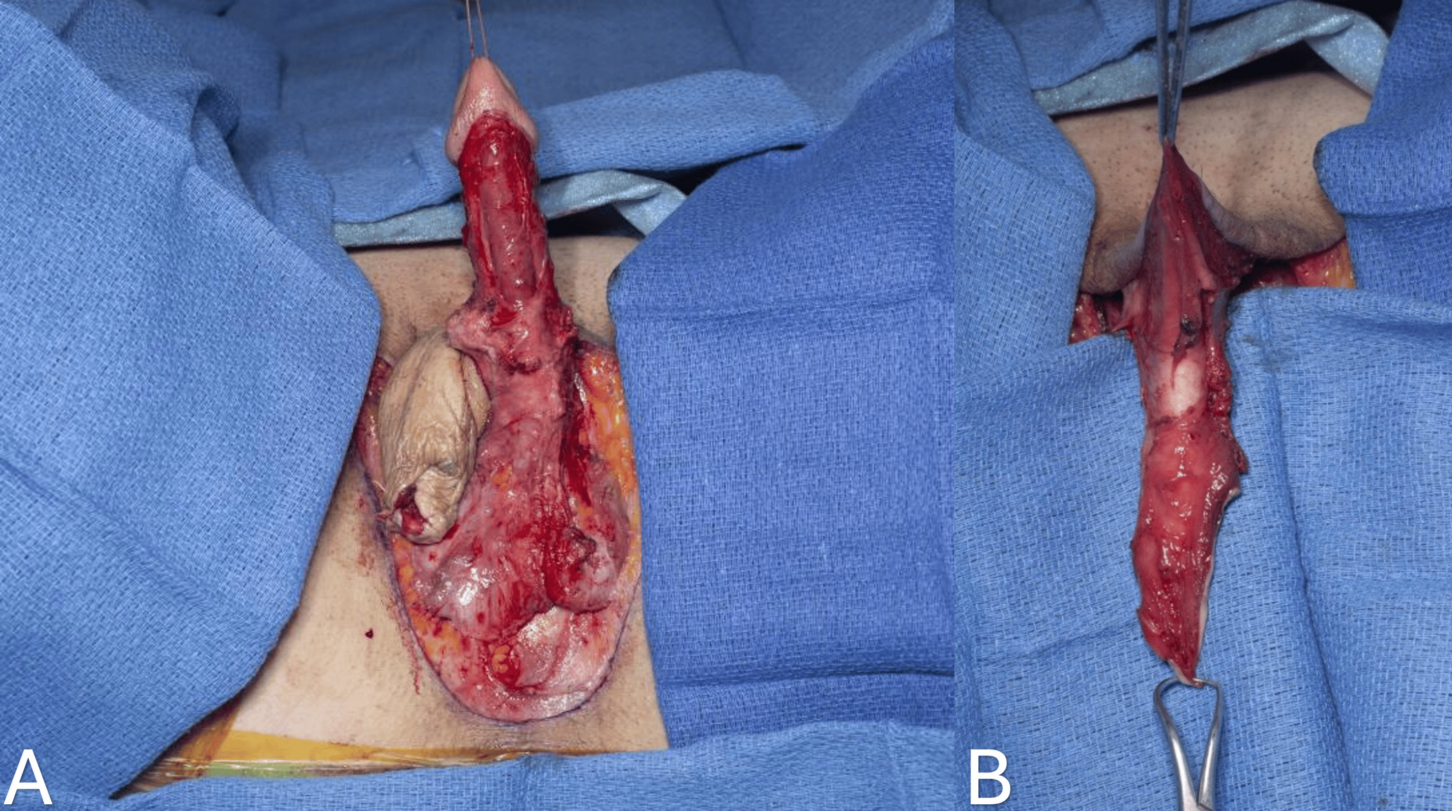
Dissection of the penile skin flap superficial to the Buck's fascia. Corpora cavernosa, corpus spongiosum, and glans penis are completely separated from the skin (A). Anteriorly inverted penile flap (B).

One of the primary objectives of vaginoplasty is to eliminate penile erectile capabilities and design a sensitive clitoris derived from the glans penis based on a pedicle of the dorsal neurovascular bundle [3]. The penile component separation begins with the degloving of the penis at the level of Buck's deep fascia to create a penile skin inversion flap. A circumferential incision is performed around the base of the glans penis, and dissection continues with Metzenbaum scissors utilizing a sharp and blunt approach. Ventrally, the incision is just proximal to the corona, while dorsally, it is about 2 cm proximal to the corona. The design of this incision allows us to preserve a 2 x 3 cm dorsal penile skin flap for future clitoral hood reconstruction. Dissection above the deep fascia is crucial to protect the dorsal penile neurovascular structures from potential injury. The anteriorly based fasciocutaneous penile inversion skin flap is then created (Figure 4).

**Figure 5:**
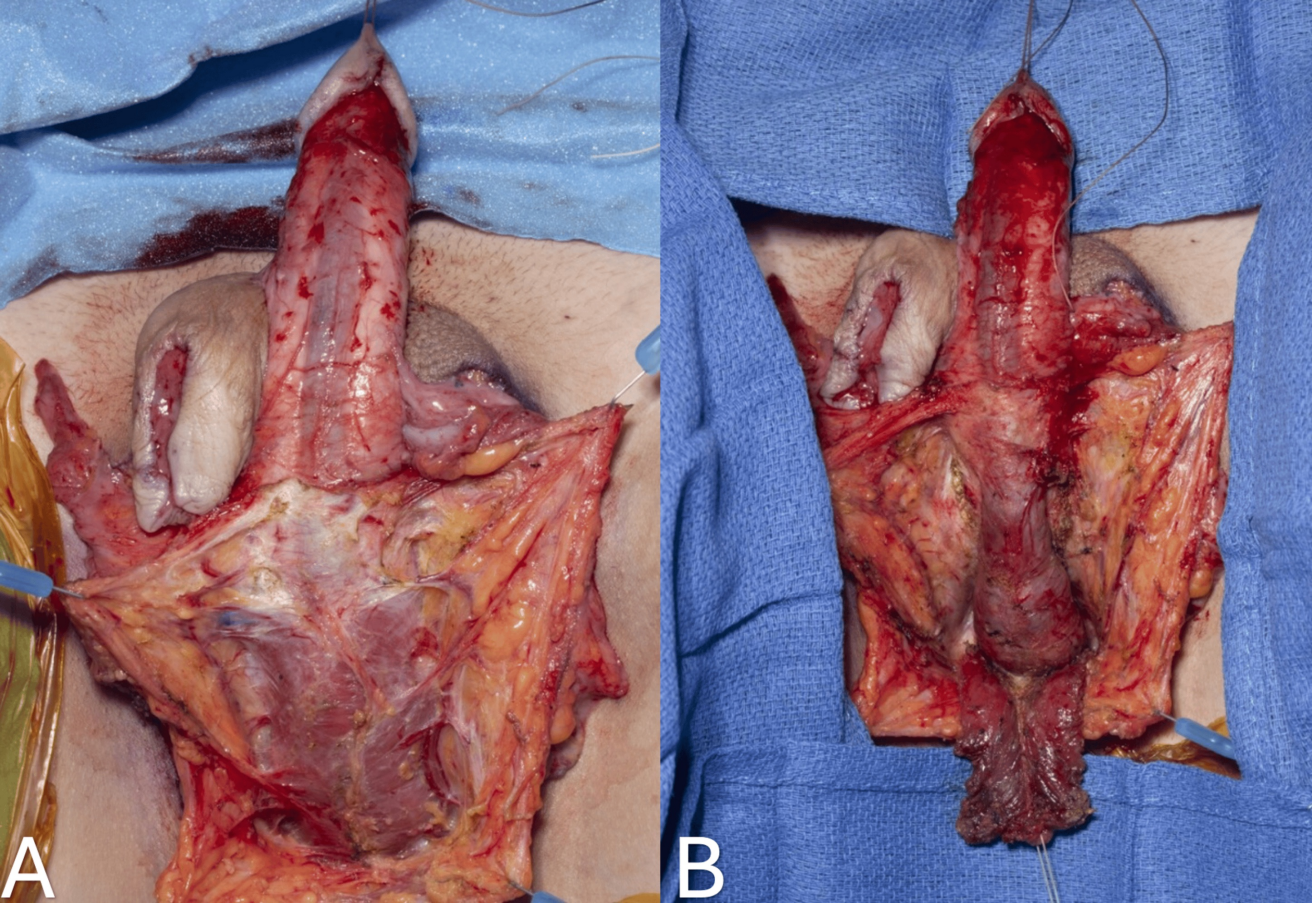
Midline dissection of adipofascial tissue and exposure of bulbospongiosus muscle (A). The bulbospongiosus muscle is dissected from the corpus spongiosum and urethra to its origin from the level of the perineal body (B).

Adipofascial tissue is dissected along the midline until the bulbospongiosus muscle is encountered. This tissue is then retracted laterally on each side, allowing the complete separation of the penile shaft from the surrounding tissue. Multiple branches of the perineal artery enter the corpus spongiosum and urethra along the lateral border. These vessels should be cauterized meticulously to avoid bleeding and postoperative hematoma. The bulbospongiosus muscle is dissected from the corpus spongiosum and urethra to its origin from the level of the perineal body (Figure 5).

It is preserved as it can be used as a pedicle muscle flap to bolster the repair of any potential rectal injury, which aligns with other studies [6,7,12]. The urethra and corpus spongiosum are dissected from the corpus cavernosa, starting from the midshaft down to the bifurcation of the corpus cavernosa. The urethra is cut, and a Foley catheter is inserted. Palpation of the Foley catheter and its balloon helps to identify the course of the urethra and prostate during the canal dissection. We maintain enough urethral length to ensure it reaches the level of the adductor tendons, which is typically where the clitoris will be positioned. 

**Figure 6:**
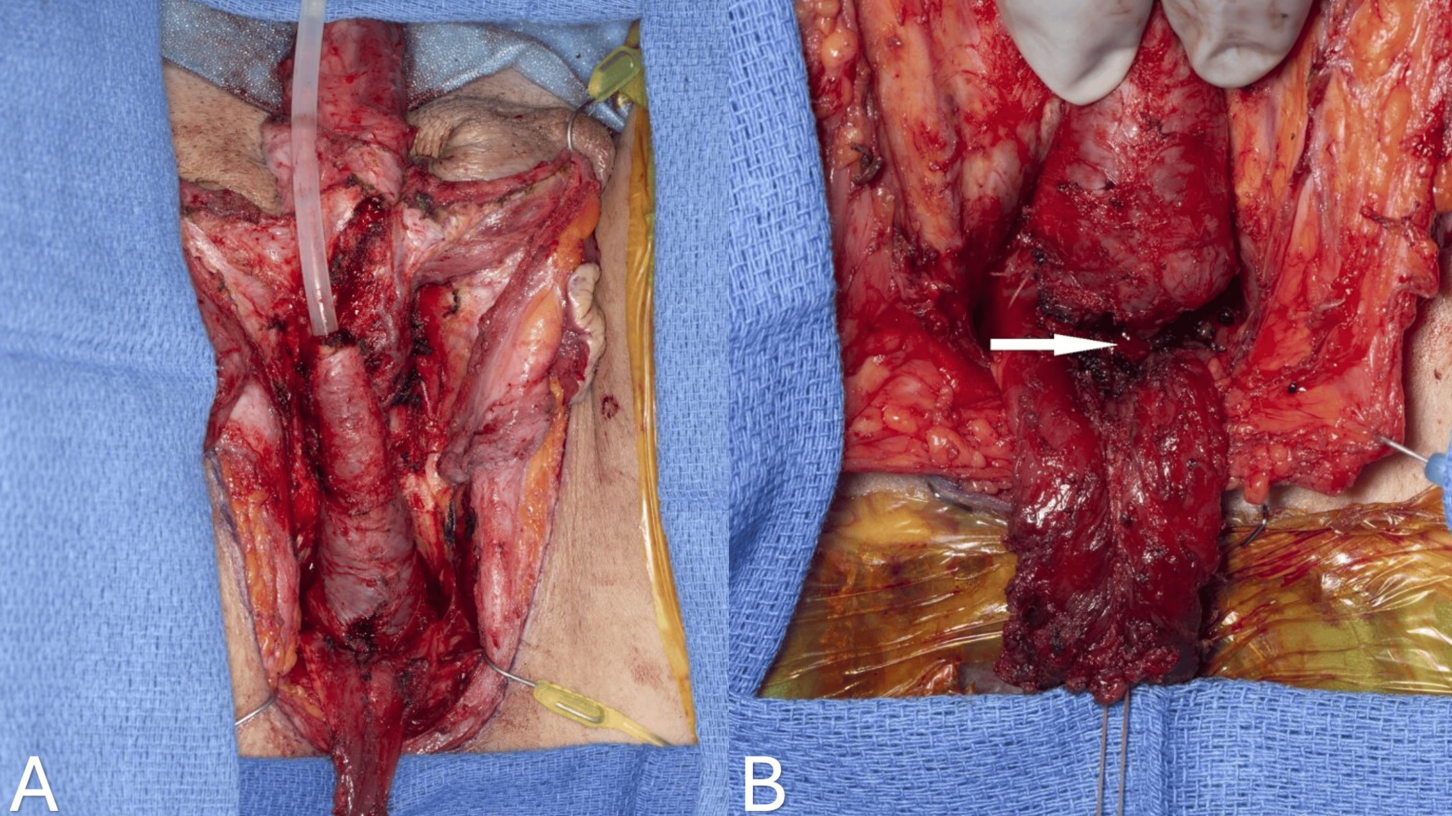
The urethra is transected to the level of the adductor longus tendon and a Foley catheter is inserted (A). Dissection of the vaginal canal between the bulbospongiosus muscle and bulb part of the urethra (B) (white arrow).

To create the neo-vaginal space, we start the dissection between the bulbospongiosus muscle and the bulb of the penis (Figure 6). Some authors prefer starting the dissection through the perineal body [3,6-8]. The dissection of the neo-vaginal canal is performed sharply and bluntly using electrocautery and vaginal dilators. The utmost care and attention are required during the dissection of the prostate from the anorectal junction. This area presents the highest risk for potential rectal or urethral injury due to the tight attachments between these structures. Dissection stays on the prostatic capsule to avoid damaging the rectum. The Denonvillier's fascia is split into anterior and posterior layers following this dissection [6,7].

Following the creation of the neovagina, there are varying degrees of pelvic muscle release, ranging from no release to multiple releasing incisions to a particular endpoint [3,7]. To expand the vaginal canal, we partially divide the levator muscle. We use progressively larger diameter dilators to widen the vaginal canal (Video 1). The neo-vaginal space is then irrigated, and hemostasis is ensured with electrocautery, bipolar electrocautery, and dressings soaked in a sterile solution of 75 mL of tranexamic acid 3 g/75 mL (40 mg/mL) in NaCl 0.9%. Following the successful dissection of the vaginal canal, the bulbospongiosus muscle can be resected and discarded. When left behind, it can create unpleasant bulging at the vulva or animation deformity. 

**Video 1:** Vaginal canal dilation

The vulvar reconstruction involves forming a neo-clitoris, adjusting the length of the urethra and position of the urethral meatus, and removing the excess penile tissue. According to Dr. Shoureshi and Dr. Dugi, the construction of the clitoral hood and inner labia depends on available penile skin, with surgeons using inner preputial shaft skin when sufficient or an extended urethral flap when penile skin is limited to achieve optimal aesthetic results [3].

In the senior author's practice, the creation of the clitoris evolved from using the penile tip to a W flap. Now, we prefer to use a small 2 x 0.5 cm flap from the dorsal corona. It allows us to create the smallest possible clitoris that still has normal sensation. The lateral ends of this incision are connected to the vertical incisions on the tunica albuginea that will be used to excise the erectile tissue of the corpus cavernosa. When designing the vertical incisions in the tunica albuginea, it is essential to place them laterally to prevent damaging the paramedian-positioned dorsal penile neurovascular bundle [7]. The erectile tissue of the corpora cavernosa is cautiously dissected with a 15-blade and electrocautery from the penile base to the separation of the crura. Following complete dissection of the penis and corpora cavernosa, the penis is excised.

We insert recombinant thrombin (RECOTHROM, Baxter) soaked hemostatic sponge containing 5,000 units into the crura and close the fascia with absorbable monofilament sutures. Following the separation of the coronal flap from the glans penis, it is folded upon itself and sutured with one 3-0 monofilament absorbable suture, resulting in the formation of a 1 cm clitoris. 

**Figure 7:**
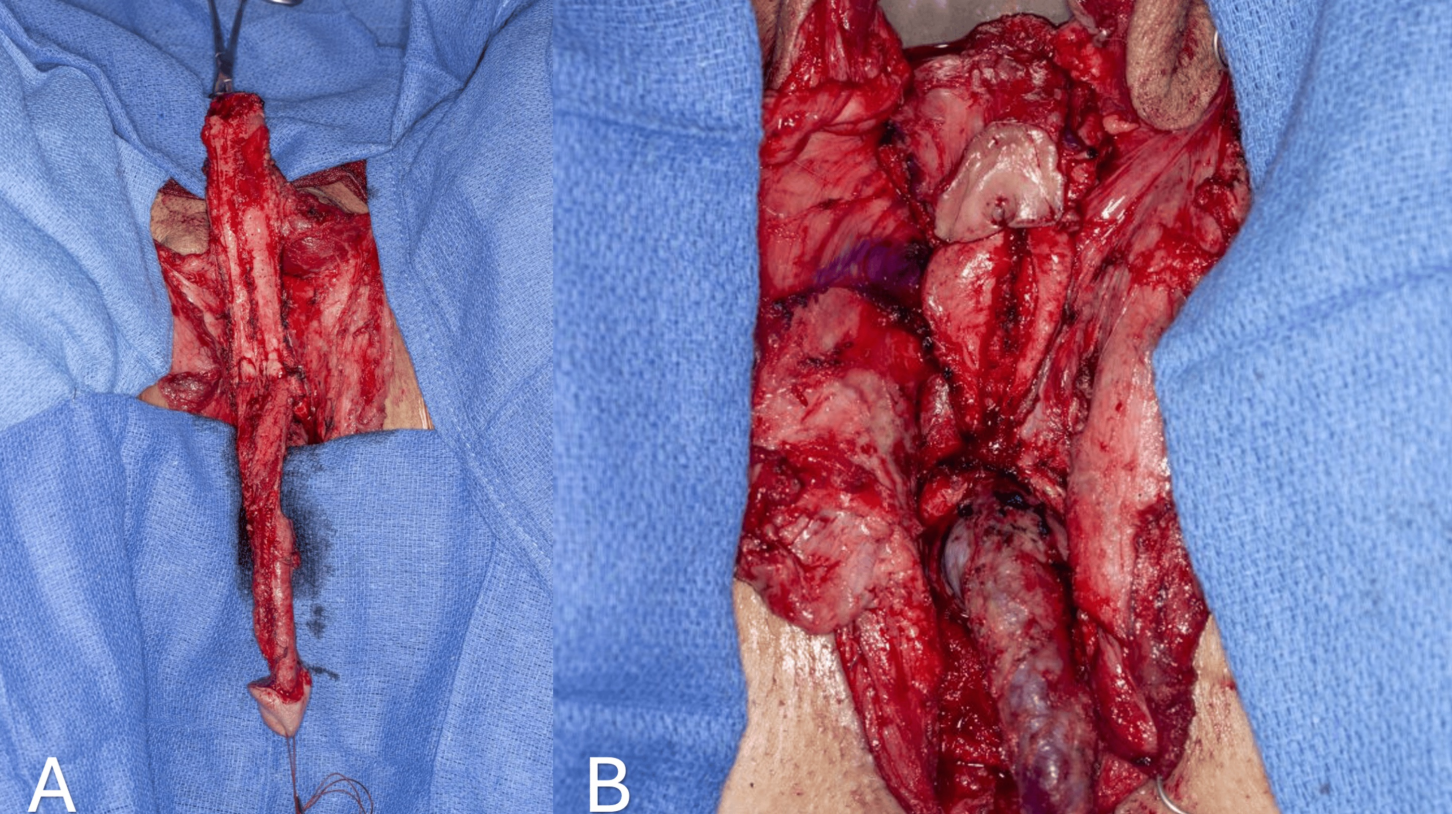
Penile dorsal coronal flap with dorsal neurovascular pedicle separated from the glans penis and corpus cavernosa (A). The neo-clitoris inset at the level of the adductor longus tendon (B).

Ultimately, the neurovascular pedicle is folded and buried in the mons pubis. Neo-clitoris is positioned at the level of the adductor longus tendon (Figure 7). Massie et al. select this same position for the neo-clitoris. The neo-clitoris receives its blood supply and sensation from the dorsal neurovascular bundles of the penis that are protected dorsally by Buck’s fascia and ventrally by the tunica albuginea of the corpora [13].

**Figure 8:**
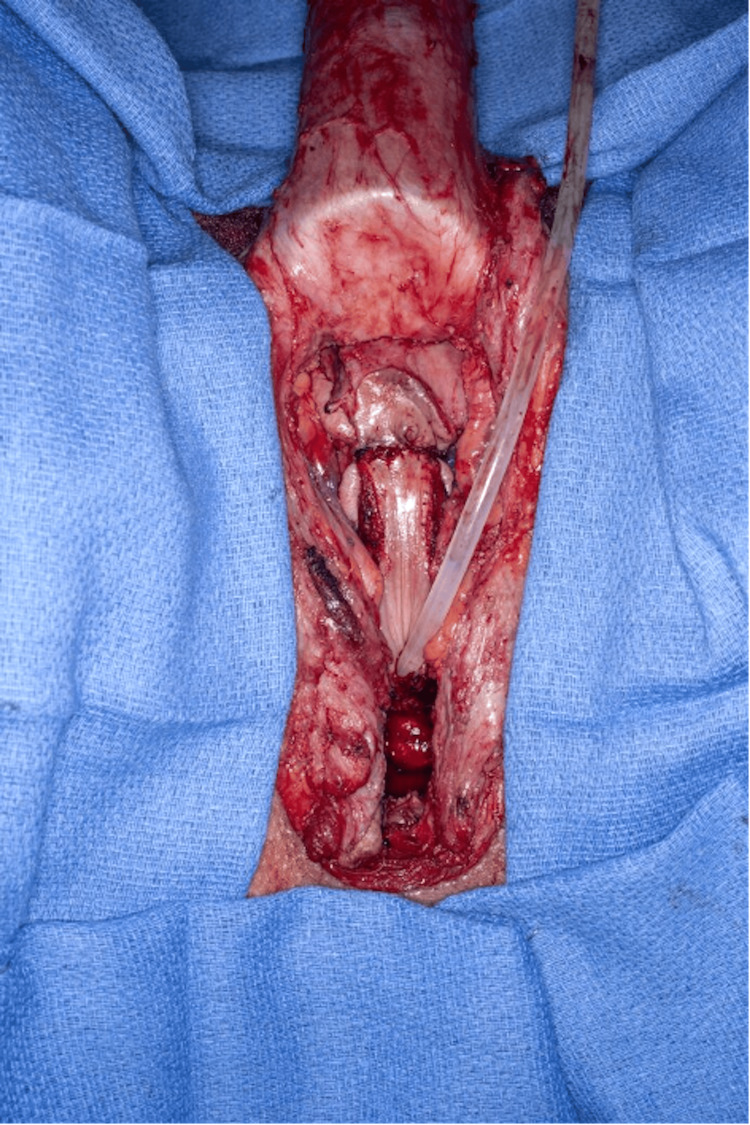
Lowered urethral meatus, transected end of the urethra, and corpus spongiosum flap secured at the base of the neo-clitoris.

Subsequently, the urethra and corpus spongiosum are split from the midline using a ventral vertical incision. The transected end is secured just under the neo-clitoris (Figure 8). Careful attention is given to ensuring the split is inferior/posterior enough to direct the urinary stream downward rather than anteriorly. The corpus spongiosum surrounding and beneath the new urethral meatus is excised to prevent engorgement during sexual arousal and to mitigate the potential for a phantom penis phenomenon.

**Figure 9:**
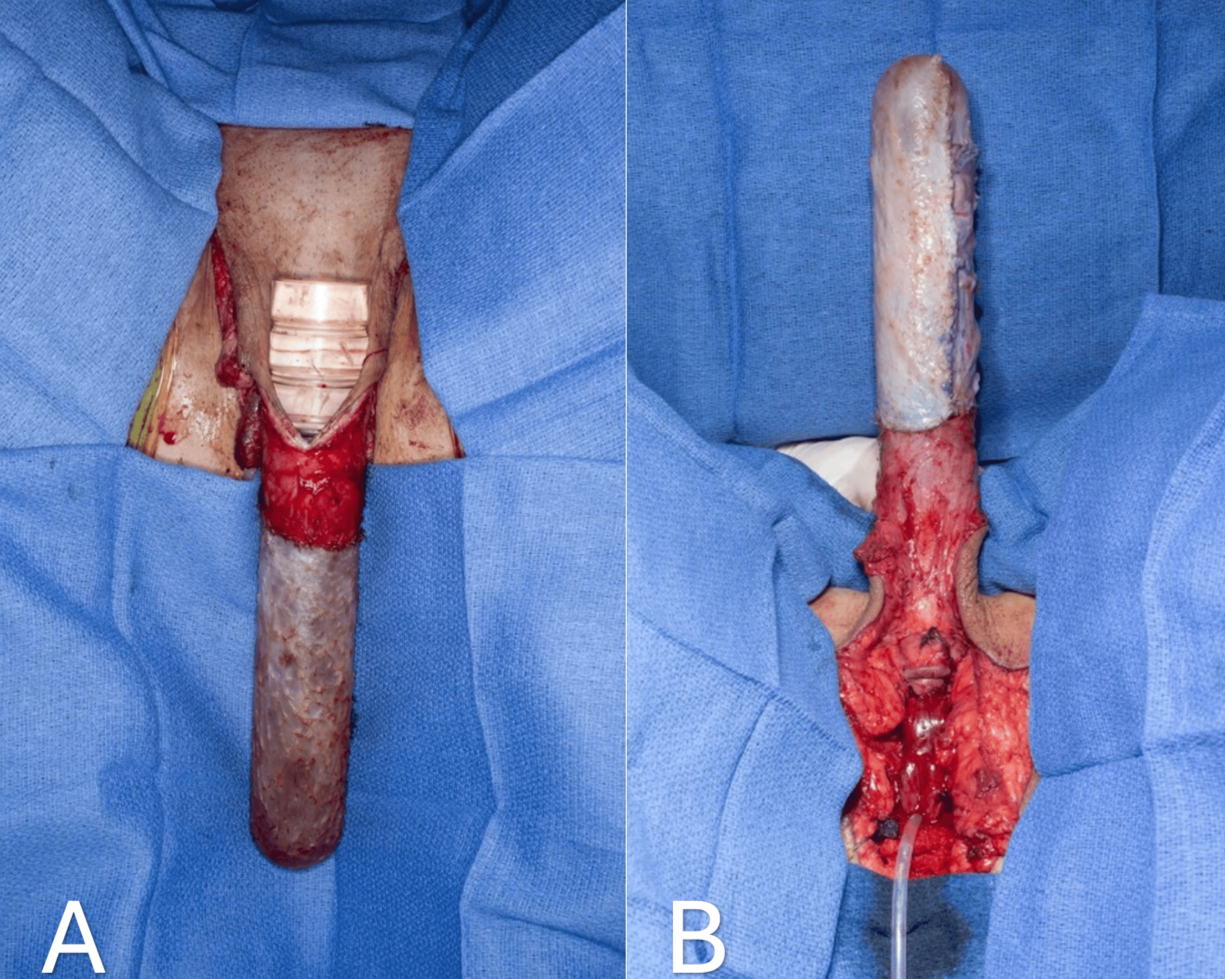
Creation of vaginal lining by connecting the tubularized scrotal skin graft to the penile inversion skin flap (A). Radially lined tunica vaginalis within the tubularized skin graft (B).

**Figure 10:**
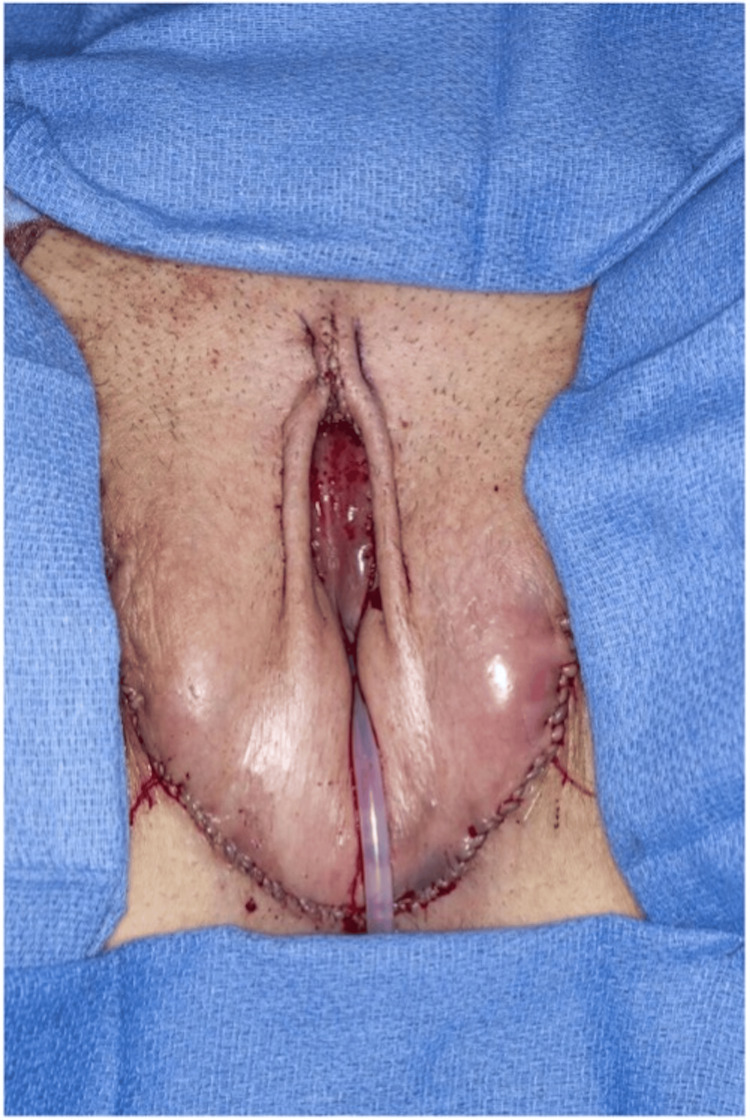
A midline longitudinal incision is made in the fasciocutaneous flap above the vaginal introitus, to deliver neo-clitoris and urethral meatus. Creation of labia minora and clitoral hood.

The penile skin flap is inverted and sutured to the tubularized scrotal skin graft with or without the tunica vaginalis to create the neo-vaginal lining (Figure 9). It is then inserted into the vaginal canal using a dilator and then packed with antibiotic-soaked vaginal packing (Bacitracin ointment 500 Units/g and metroNIDAZOLE 1 % gel (METROGEL)). Some studies employ fibrin glue or internal sutures to adhere the graft and skin flap to the underlying tissue [2,3,7]. We do not routinely use fibrin glue or internal sutures to secure the vaginal lining. A midline longitudinal incision is made in the fasciocutaneous flap above the vaginal introitus, and neo-clitoris, urethral meatus, and Foley catheter are delivered through this incision. The clitoral hood and labia minora are created by suturing the lateral edges of the urethral flap and small dorsal penile skin flap to skin edges created by midline incision (Figure 10). The lateral edges of the anteriorly based fasciocutaneous flap are advanced toward the groin crease incisions, forming labia majora.

Dressings and postoperative care

**Figure 11:**
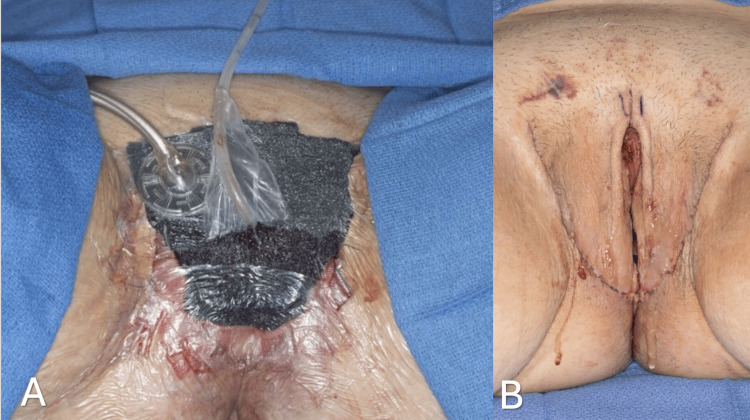
Negative pressure dressings are applied over the surgical area (A). One-week postoperative visit after wound vac dressings, vaginal packing, and Foley catheter is removed (B).

The senior author places a Penrose drain along the posterior aspect of each lateral groin incision, securing each in place with a loosely applied 3-0 non-absorbable monofilament suture. Liposomal bupivacaine (Exparel; Pacira Pharmaceuticals, Inc., Parsippany, N.J.) is injected around the incisions. Xeroform (CURAD) is carefully placed in the vulvar vestibule surrounding the Foley catheter (Figure 11). Negative pressure dressings are then applied over the surgical area. Patients are admitted to the general care floor. Perioperative antibiotics are continued for 24 hours. They are encouraged to start ambulation on postoperative day one. Prophylactic anticoagulation with subcutaneous injection of Enoxaparin (LOVENOX) 40 mg is administered while inpatient. Patients are usually discharged on postoperative day three. The wound vac dressings, vaginal packing, and Foley catheter are removed during the first postoperative visit to the clinic in one week. Moreover, the graft status is checked on postoperative day seven, the same day the vaginal packing is removed. At the same time, dilation teaching is performed, and patients begin to do vaginal dilations on the same day according to our protocol.

## Discussion

“Gender-affirming vaginoplasty” represents a complex, multifaceted surgical procedure. Traditional PIV uses penile skin to line the neo-vaginal canal, but this alone often falls short of providing complete coverage [2,3]. As a result, scrotal skin grafts are routinely necessary. In cases where scrotal skin grafts are insufficient, techniques rely on full-thickness skin grafts from the groin area, intestinal segments, and/or urethral flaps [3,6,11,14,15]. However, these approaches can result in additional scarring. Our technique differs by preferentially utilizing tunica vaginalis as a supplementary graft when necessary. The percentage of the patients in which the senior author has incorporated tunica vaginalis into the vaginal canal comprises 51.6%. Tunica vaginalis as a supplementary graft is particularly noteworthy, as it can be easily harvested during orchiectomy without introducing additional scarring.

Our management of the bulbospongiosus muscle also differs from some established techniques. Rather than immediate excision, we initially preserve this muscle, allowing for its use as a pedicle muscle flap to reinforce any potential rectal injury repair if needed [6,7,12].

The method we employ to create the neo-vaginal space represents another departure from some common approaches by initiating dissection between the bulbospongiosus muscle and the bulb of the penis rather than through the perineal body. The latter can damage the dominant blood supply to the bulbospongiosus muscle, making it impossible to use in the future. 

Significant variation has been studied in how surgeons create the neo-vaginal space [7]. Some surgeons utilize the Lowsley retractor to mobilize the prostate into the surgical field. However, the anorectal junction is closely associated with the apex of the prostate. It is at risk of injury, especially when the Lowsley retractor pulls it and the prostate further up into the wound [3]. We use the Foley catheter to define the membranous and prostatic urethra; this technique does not require mobilization of the prostate. Frequent palpation of the Foley catheter and balloon and digital rectal examination are helpful to guide a safe dissection. Additionally, a rectal dilator is also used to assist with pelvic dissection.

Techniques of the resection of the corpora cavernosa may differ between surgeons [7,15]. Dissection of the corpora cavernosa can be at the bony projection or the crus [7]. While a technique involving the removal of the corpora cavernosa at their attachments to the pubic bone exists, its clinical benefits lack objective supporting data [2]. 

According to Claes et al., most surgeons form the neo-clitoris using the dorsal portion of the glans of the penis in a W pattern [16]. Other authors create the neo-clitoris drawing a zig-zag, M, or V pattern [12]. Our approach to clitoris formation has evolved over time, from the penile tip to a W flap, culminating in the use of a small 2 x 0.5 cm flap from the dorsal corona. This method aims to create the smallest possible clitoris that still retains normal sensation, offering improved aesthetic outcomes. 

Contrary to other methods, our technique does not routinely employ fibrin glue for graft adherence. This decision is because fibrin glue is a hemostatic agent. While initially, it may improve the stability of the graft, it can form a barrier between the skin graft and the wound bed, which may adversely affect the graft's take and survival. Finally, our postoperative care protocol incorporates modern wound management techniques, including using negative pressure dressings and injecting liposomal bupivacaine around incisions. These approaches may contribute to improved healing, shorter postoperative stays, and enhanced patient comfort during recovery.

## Conclusions

As the field of gender-affirming surgery continues to evolve, ongoing refinement of surgical techniques, guided by clinical experience and research, will be crucial in optimizing care for transgender individuals seeking vaginoplasty. To achieve optimal outcomes, a precise step-by-step approach is imperative. Advancements in research and technology continually introduce nuanced refinements to surgical techniques. These may include innovative practices such as folding the neurovascular flap, preserving the bulbospongiosus muscle if required, harvesting the tunica vaginalis, applying tranexamic acid, utilizing negative pressure dressings, and other evolving strategies, all of which collectively may contribute to enhancing the likelihood of a successful surgical outcome.
